# Occurrence of Surface Active Agents in the Environment

**DOI:** 10.1155/2014/769708

**Published:** 2014-01-16

**Authors:** Ewa Olkowska, Marek Ruman, Żaneta Polkowska

**Affiliations:** ^1^Chemical Faculty, Gdansk University of Technology, G. Narutowicza 11/12 Street, 80-233 Gdansk, Poland; ^2^Faculty of Earth Sciences, University of Silesia, Będzińska 60 Street, 41-200 Sosnowiec, Poland

## Abstract

Due to the specific structure of surfactants molecules they are applied in different areas of human activity (industry, household). After using and discharging from wastewater treatment plants as effluent stream, surface active agents (SAAs) are emitted to various elements of the environment (atmosphere, waters, and solid phases), where they can undergo numerous physic-chemical processes (e.g., sorption, degradation) and freely migrate. Additionally, SAAs present in the environment can be accumulated in living organisms (bioaccumulation), what can have a negative effect on biotic elements of ecosystems (e.g., toxicity, disturbance of endocrine equilibrium). They also cause increaseing solubility of organic pollutants in aqueous phase, their migration, and accumulation in different environmental compartments. Moreover, surfactants found in aerosols can affect formation and development of clouds, which is associated with cooling effect in the atmosphere and climate changes. The environmental fate of SAAs is still unknown and recognition of this problem will contribute to protection of living organisms as well as preservation of quality and balance of various ecosystems. This work contains basic information about surfactants and overview of pollution of different ecosystems caused by them (their classification and properties, areas of use, their presence, and behavior in the environment).

## 1. Introduction

Surface active agents (SAAs, surfactants) are a group of compounds with specific chemical composition of their molecules (one part soluble in polar medium: hydrophilic and second in nonpolar medium: hydrophobic). The main classification of surfactants is based on charge of hydrophilic part of their molecules: cationic, anionic, and nonionic compounds [1].

Occurrence of polar and nonpolar parts in the SAAs molecules gives them special properties against different medium. Surfactants are characterized by tendency to absorb at different types of surfaces or interfaces. Another property of the SAAs is ability to association in solution and formation of micelles [2]. During the process of formulation micelles surface active agents are adsorbed at boundary phases to remove hydrophobic parts from water to reduce energy of system [3].

Due to the specific chemical structure of surfactants molecules they are applied in different areas of human activity. During formulation of households or industrial products compounds from the group of surfactants are used because their presence leads to improving efficiency of the following processes:

- wetting/waterproofing,
- de- or foaming,
- de- or emulsification,
- dispersion or flocculation of solids particles in liquid phases,
- solubilization of non-/sparingly soluble reagents in solvents,
- increase or decrease of viscosity of solution phases [4].

In Table 1 general areas of surfactants application are presented.

In 2008 the annual global production of surface active agents was about 13 million metric tons. The expected growth of production of that class of compounds is forecasted as 2.8% annually to 2012. Approximately 65% of total production corresponds to compounds classified as anionic surfactants [3], second and third places in global production corresponds to nonionic and cationic compounds, respectively.

After use, compounds from the group of surfactants are emitted to various elements of the environment (gas, liquid, and solid phases), where they can undergo numerous physical and chemical processes. Therefore, the specific properties of those chemical compounds cause increasing their mobility and unrestrained circulation in the environment. Those processes might significantly contribute to disrupting the water cycle within various ecosystems; hence, it is essential to obtain answers to what levels of concentrations surfactants are present in the environment. Investigation of environmental fate of surface active agents can help increase level of knowledge about pollutants migration pathways and better protect living organisms or different ecosystems. In recent years the interest of SAA has increased and scientists have started to estimate the possible effects of those compounds on the environmental balance [5, 6].

In this paper, basic information about surface active agents (classification, their properties, and areas of use) and their fate after discharging to wastewater treatment plants are mentioned. The brief review of sorption and degradation processes of surfactants in water systems is presented. Such information can be useful during investigation of their presence and behavior in environment. Moreover, the analytical protocols used for determining total concentration of surfactants or individual analytes from particular group of SAA in environmental samples are described. This work contains overview of contamination of different ecosystems caused by surfactants (also research data of levels of SAA in atmospheric deposition samples collected in urban and nonurban areas). Possible impact of compounds from the group of SAA on biotic and abiotic elements of the environment (especially as a result of their occurrence in atmospheric waters) is presented.

## 2. Emission Pollutants into the Environment and Their Fate

Surface active agents are one of the most common applied compounds in industrial, agricultural, and household activities and after use a huge number of surfactants (and/or their degradation products) are discarded to wastewater-treatment plants (WWTP). In nonurban areas (there is no WWTP) wastewaters that contained various classes of surface active agents are discarded directly to surface waters and they might be dispersed into different elements of environment. In wastewater treatment plants compounds from group of SAAs are completely or partially removed by a combination of different processes (mainly by sorption and aerobically biodegradation) and their degradation pathways were investigated [7, 8].

Chemical compounds from this group are degraded during secondary treatment processes and under optimized conditions about 90–95% of initial SAAs concentration contained in influent streams can be eliminated (depending on efficiency of WWTPs) [9]. In should be noticed that considerable part of pollutants is removed as sewage sludge (from 15% to more than 90%) [10, 11]. Moreover, some surface active agents can be transformed to more toxic degradation products (e.g., degradation products of compounds from the group of alkylphenol ethoxylates (APEO)).

After appropriate processes in WWTP effluents and sewage sludge, in which different types of surfactants or their degradation products (several *μ*g/L or g/kg) can occur, are discharged into surface waters or used as fertilizer in agricultural areas, respectively. Such practices lead to emission of surfactants and their metabolites into different parts of the environment (soils, ground waters, surface waters, and living organisms) [9, 12]. In recent years the amount of literature data concerning on surfactants occurrence and their concentration in environmental samples has been markedly increased.

If compounds from the group of SAAs are present in water ecosystems, they can also undergo sorption and aerobic/anaerobic degradation processes. Those processes are responsible for elimination and transport of pollutants to water systems. Sorption processes inhibit also degradation of chemical compounds because their bioavailability can be reduced. In Table 2 data concerning surfactants sorption process in environmental samples are presented. Research allows observing relationship between higher salinity of water samples and higher sorption percentages for compounds from the group of LAS onto suspended solids (as calcium and magnesium salts).

Generally, the higher concentrations of less polar compounds from the group of surfactants (e.g., C_13_LAS, NP, and NPE_1-2_O) were observed in sediment or suspended solid samples. The higher concentrations of more polar compounds (e.g., C_10_LAS, short-chain SPC, and NPEC) were observed in the dissolved form [13–15]. Sorption process is relative to hydrophobic nature of compounds:

- more polar anionic SAAs were estimated in the dissolved phases;
- less polar cationic and nonionic SAAs were estimated in the particulate phases (their transport will be associated with suspended solids) [15].

Also other environmental factors like pH, salinity, carbon, or clay content of the particulate phase can have influence on sorption processes [8, 16].

Degradation of surface active agents caused by microbiological organisms (biodegradation) is the primary transformation taking place in different ecosystems to reduce impact on living organisms. Biodegradation is an important process not only in wastewater treatment plants but also in the environment. During this process microorganisms are able to utilize surfactants as substrates to produce energy and nutrients or cometabolize them by microbial metabolic reactions. There are many factors (e.g., chemical structure of analytes, physiochemical parameters of ecosystems like temperature, light, and salinity) that affect the efficiency of biodegradation of compounds from the group of surfactant in the environment. Most of compounds from this group can be rapidly degraded by microorganisms in ecosystems in the presence of oxygen (according to the current legislation), while some of them (e.g., LAS, DTDMAC) may be persistent under anaerobic conditions [8, 15, 17]. In Table 3 research data from aerobic degradation assay carried out on target compounds are presented.

Cationic surfactants are aerobic biodegradable compounds that characterized different degradation mechanisms. The degradation pathway for alkyl trimethyl or dimethyl ammonium compounds (TMAC and DMAC) is initialed by N-dealkylation and followed by N-demethylation reaction (trimethylamine, dimethylamine, and methylamine were identified as the intermediates of alkyl trimethyl ammonium salts in activated sludge) [18]. Cationic surfactants containing a quaternary ammonium (QAC) often have strong biocidal properties [19]. The length of alkyl chain has an important role in the fate and biological effects of these compounds in the environment. The aerobic biodegradability of compounds from the group of QACs decreases with the number of nonmethyl alkyl groups (e.g., R_4_N^+^ < R_3_MeN^+^ < R_2_Me_2_N^+^ < RMe_3_N^+^ < Me_4_N^+^; Me—methyl group) and substitue them with a benzyl group [20, 21]. The biodegradation was not observed for ditallow dimethyl ammonium chloride (DTDMAC) in anaerobic screening assay and this compound was replaced by diethyl ester dimethyl ammonium chloride (DEEDMAC) [22].

Analytes from the group of LAS contained longer alkyl chains and benzene group in external position is more susceptible to biological degradation. Mono- and dicarboxylic sulfophenyl acids (SPC), LAS biodegradation intermediates having an alkyl chain length of 4 to 13, are formed during the following stages: *ω*-oxidation of the alkyl chain terminal carbon, successive *β*-oxidation, and further desuphonation [7, 23–26].

For example, it can be observed that in surface waters primary degradation (compounds that lost their chemical structure and properties) of compounds from the group of LAS is completed after 4 days; the average half-life of some analytes is 10–24 h and 56–90% of mineralization can be finished from 7 days up to 30 days [8]. Compounds from the group of fatty alcohol sulphates (AS) undergo rapidly primary and ultimate biodegradation process under aerobic and anaerobic conditions [7, 8, 21]. The degradation process involves the enzymatic cleavage of the sulphate ester bonds to give mixture of inorganic and organic compounds (sulphate and fatty alcohol). During further stages, alcohol is oxidized to aldehyde and next to fatty acid (*β*-oxidation pathway) [27]. Biodegradation of compounds from the group of SDS was reported in Antarctic coastal waters with half lives of 160 to 460 h [28]. Removal efficiency in WWTP of homologues from the group of SAS was estimated from 64 to more that 99% (due to a combination of degradation in the active sludge unit (84%) and sorption processes onto sludge (16%)). The degradation process is depending on length of alkyl chain in SAS homologues due to decreasing solubility for longer chains in polar medium (shorter alkyl chain = higher degradation percentage). In addition, long alkyl chain of SAS compounds is characterized by a about three times higher tendency to sorption onto sludge surfaces compared with short chain compounds [29, 30]. For other anionic surfactant like AES removal efficiency from influent water was achieved about 98% [31].

Degradation process of nonionic surfactants was investigated mainly using compounds from the group of nonylphenol ethoxylates. Their primary biodegradation is relatively fast (from 4 to 24 days) and their mineralization is typically from 50 to 80% [32–34]. Degradability of NPEO compounds with lower molecular weight is easier than that with higher molecular weight [35]. It was confirmed that during biodegradation process the ethoxylated chain becomes progressive shorter as a result of hydrolysis and next oxidations reactions, leading to short-chain NPEs with one or two ethoxylate groups [32, 36]. Besides, oxidation reaction of the ethoxylated chain can occur more often than hydrolysis (NPECs are the most often found metabolites: 69–98%) and NPEC compounds can be degraded to NPE_2_C [33–35]. Alkylphenol diethoxycarboxylates can be also formed as a consequence of *ω*-oxidation and later *α*, *β*-oxidation reaction of the alkyl chain [34]. In different types of environmental samples were detected metabolites of NPEO compounds (NP, NPE_1-2_O, and NPEC) [8, 37, 38]. Degradation of compounds from the group of alcohol polyethoxylates (AEO) was also investigated. Biodegradation processes can be more efficient for analytes with shorter alkyl and/or ethoxylated chains. In this process fatty acids and polyethylene glycols (PEG: slower biodegradation with production of carboxylic acids) can be formed as a consequence of central cleavage of molecule and undergo *ω*-oxidation and later *α*, *β*-oxidation reaction of the alkyl chain [39, 40]. Branched compounds from the group of AEO are characterized by slower degradation and it involves *ω*-oxidation and successive *α*, *β*-oxidation reactions of the alkyl chain [24].

Therefore, surfactants occurred in water systems can be degraded easily (half-lives from hours to few days) depending on their properties and environmental parameters. Compounds from the group of SAA can undergo such processes like attachment to suspended solids and accumulation in sediments. In conditions with lack of oxygen (starting in depth of few cm) surfactants can be only degraded by anaerobic pathways. Generally, in anaerobic condition processes are slower or they are not observed (e.g., DTDMAC) and pollutants occurred longer in sediment [8, 13]. However, during laboratory experiments acceptable degradation percent of LAS has been observed with use of anoxic marine sediments (up to 79% in 165 days). The following stages of anaerobic degradation pathway for LAS have been reported: initial reaction metabolites (generated via the addition of fumarate); their biotransformation into sulfophenyl carboxylic acids, and progressive degradation by *β*-oxidation reactions [41–43].

## 3. Levels of Surfactants in Different Part of Environment

For different reasons, it is important to detect, identify, and monitor levels of surfactants in aquatic environments. Prior analytics of pollutants from the group of SAAs in environmental samples pose new challenges. The searching for new tools and new sources for obtaining information about degree of pollution different environmental compartments is dictated by toxicological considerations, the desire to increase the accuracy of the description of the environment, and the study and protection of aquatic ecosystems balance. It is imperative to have the appropriate analytical tools (standard analytical methodologies or their modification) in order to monitor the presence of surface active agents in various environmental samples. The determination of SAAs in such samples causes a lot of problems, mainly because of [1, 6]

- the complex composition of environmental samples—interfering components which increased or reduced the identified levels of analytes,
- the low concentrations of individual surfactants in such samples,
- the diverse chemical structures of surfactants—moreover commercially available surfactants are mixtures containing twenty or more individual compounds,
- the amphiphilic nature of surfactants (a consequence of their chemical structure),
- the limited availability of commercial standard solutions of surfactants (also isotope-labelled analytes).

The complex and frequently variable matrix composition of environmental samples and the trace levels of SAAs mean that suitable isolation and/or preconcentration techniques have to be applied at the sample preparation stage. As a consequence of amphiphilic nature of surfactants molecules during preparation stage an internal standard has to be added to the sample before the solvent extraction (for estimating the losses of analytes), which problematic because of lack of available commercial standards. Moreover, the analytical methodologies enabling the determination of a wide range of SAAs present at different levels in environmental samples should be validated against certified reference materials. Nowadays, only liquid reference materials are available on the market; they can be used to validate methodologies for determining total contents of ionic (cationic or anionic) and nonionic surfactants. On the other hand, there are no reference materials suitable for validating entire analytical procedures. Those problems have influence on quality control and quality assurance of measurement results and they might cause difficulties in obtainment of the reliable analytical information [44, 45]. Currently, despite an increase in the amount of information about the concentration of compounds of the group of SAAs in environmental samples, the knowledge about degree of pollution caused by surfactants is too low and it is still impossible to determine how they can affect the diversity of ecosystems.

The determination of total concentration of ionic and nonionic surfactants in different types of liquid/solid samples can be carried with use of standard analytical methodologies (including liquid-liquid extraction/solid-liquid extraction or Soxhlet extraction, resp.). Cationic surfactants can be determined as sum of substances that form ion pairs with disulfine blue (DiSB) and isolated with use of appropriated extraction technique. Next, the absorbance of the organic extract is measured spectrophotometrically [46]. Total concentration of compounds from the group of anionic surfactants can be evaluated as substances which react with methylene blue (MB). At final determination stage spectrophotometrical technique can be employed to measure total concentration of anionic surfactants in solvent extract [47]. The total concentration of nonionic surfactants can be determined with use of the same analytical technique as before but with the application of different reagents [48].

For determining the individual surfactants belonging to different classes of chemical compounds should be applied modification of the available analytical procedures with use of isolation or/and preconcentration techniques at sample preparation stage (e.g., liquid-liquid extraction; solid phase extraction; solid phase microextraction) and chromatographic techniques coupled with different detection systems (e.g., GC-MS or HPLC-CD, HPLC-MS) at the final determination stage. Intensive researches in this direction are carried out in a few research centers located only in Spain, Germany, USA, China, and so forth. Gas chromatography (GC) is limited to volatile analytes and this requirement meets only low molecular mass nonionic surfactants (contain low number of ethoxylated groups). This technique is suitable during determination of concentration of other nonionic and anionic after derivatization processes with specific agents. In the literature data not mentioned about application of GC technique to separate cationic SAAs. Presently, high-performance liquid chromatography (HPLC) is the most often used analytical technique during analysis of surface active agents from all classes compounds (their homologs, oligomers, and isomers) in environmental samples. In most cases, derivatization processes of analytes are not necessary, because LC technique is suitable for determining level of low-volatility analytes with large molecules. It gives the possibility of excluding this operation from analytical procedures and simplifies them—due to the Green Analytical Chemistry (GAC) concept. An undoubted advantage of HPLC technique is that surfactants levels can be measured in a very short time. For determining the concentration of individual analytes from group of surfactants in appropriate prepared solvent extracts the following types of detectors can be used: fluorescence (FLD), ultraviolet (UV), conductometric (CD), mass spectrometry (MS) or their combinations [1, 6]. Mass spectrometer is universal detector, which can be used in qualitative and quantitative determination of wide range of trace analytes in single analysis. But this detection technique has several disadvantages (high cost of equipment and its operation; highly qualified staff; high purity of reagents). According to these drawbacks if it is possible, other detector should be used. For example, ion chromatographer coupled to conductometric detector can be applied to determinate individual ionic (cationic and anionic) surfactants in solvent extracts, for example [49]. To investigate other groups of SAA compounds (with chromophores groups in their molecules) in appropriate extracts can be involved ion chromatographer coupled to UV detector (compounds from the group of LAS—data under preparation by our research group). Other analytical tool used to determine surface active agents coupled to IC equipment is evaporative light scattering detectors (ELS). This device can detect almost all relatively nonvolatile analytes and is insensitive for gradient condition of analysis [50–52].

Analysis of the literature data confirmed that surface active agents are presented in various elements of the environment, but researchers focused mainly on determination of levels of anionic and nonionic analytes. Thus, due to the very limited literature data on the determination of cationic SAAs, which are characterized by higher toxicity to living organisms and undergo sorption onto solid surfaces. Additionally, commercial available surfactants are mixtures of various homologues and/or isomers and their determination in environmental samples becomes more problematic. Those aspects indicate the need to develop new methodologies with the use of more selective and specific analytical tools.

The ranges of concentrations or mean values of compounds from different groups of surfactants determined in solid and liquid environmental samples are presented in Table 4. Easily, it can be noticed that there is lack of research describing the problems of determining surface active agents (both total concentration or contents of individual analytes) in aerosol samples, atmospheric precipitation, and atmospheric deposits samples (e.g., dew, hoarfrost, and fog). Similarly the process of SAAs transformation in the snowpack deposited on the ground as well as the transfer of the deposited SAAs to surface waters has not been satisfactory recognized yet with hardly any publications on this topic. Studies on degree of environmental contamination caused by surfactants should be analyzed in mentioned type of samples, because atmospheric deposition is considered as a major source of various pollutants.

Accordingly, for the first time the total concentration of various groups of SAAs has been determined in atmospheric deposits samples collected during different seasons on Polish territory (Table 4—section “ATMOSPHERIC WATERS”). To fulfill this research aim, known analytical procedures (with modification) were applied. Next step in investigation of surfactants fate in the environment should be determination of their individual levels in collected samples with the use of appropriate analytical protocols.

It should be noticed that surface active agents also can be derived from natural sources, particularly from phytoplankton activities. The presence of those organisms can be observed in the surface of the euphotic zone, where chemical exchange with the atmosphere is possible. Such processes may have significant influence on the boundary between the hydrosphere and the atmosphere (this aspect will be presented in further part of paper) [53, 54].

## 4. Impact of Surfactants on Living Organisms

A fraction of surfactant from influent stream can be emitted to the environment via wastewater treatment plant effluent discharge into surface waters. In aquatic ecosystems amount of compounds from the group of SAAs can be reduced by different processes (dilution, bio- and photodegradation, and sorption to suspended solids and to sediments [55]. Application of sewage sludge (containing surfactant and other pollutants) on agricultural lands as fertilizers has an impact on terrestrial living organism and plants [8].

Cationic SAAs, because of positive charge of their molecules, are strongly sorbed to the negatively charged solid surfaces of sludge, soil, sediments, metals, plastics, and cell membranes (susceptibility to accumulation and bioaccumulation). Cationic surface active agents exhibit specific properties that may prevent (or retard) the growth or cause mortality of microorganisms. These properties allows for appling them as disinfectants and antiseptic agents in different products. On the other hand, the occurrence of cationic surfactants in aquatic ecosystems is very dangereous for aquatic organisms or, in case of humans, may cause irritation or burns to the skin, eyes and respiratory system [8, 56]. Anionic compounds can be accumulated in aquatic organisms and interact with their cell membranes, proteins, and enzymes. It causes the disturbances of their biological functions, cell lysis, or even death. Additionally, decreasing surface tension properties of anionic surfactants makes easier migration processes of other toxic pollutants into living organisms [57–59]. The most often applied group of nonionic surfactants (nonylphenol ethoxylates and octylphenol ethoxylates) undergoes a quick degradation in wastewater treatment plants into short-chain alkylphenol ethoxylates and carboxylated derivatives. Nonionic compounds undergo bioaccumulation in aquatic organisms and cause chronic toxicity to them. Moreover, it should be noticed that some products of APEO degradation are dangerous also due to their estrogenic properties [60]. The cationic and nonionic surfactants can have influence on sorption processes and transport of some pharmaceutical compounds in living organisms, potentially reducing the rate of their migration through the subsurface [61].

Some compounds from the group of surfactants have a low biodegradability or their degradation products are more toxic than initial compounds or even become endocrine disruptor compounds (EDC). The toxicity properties of compounds from the group of surfactants can be used to estimate their environmental risks. In Table 5 the literature data of toxicity for different classes of surfactants to different test organisms are collected.

Chemical compounds (EDC) that occurred in the environment can present disruption properties of normal functioning of hormonal system in living organism [62]. In 1938 for the first time data about recognition of estrogenic properties of *p*-*n*-alkylphenol were published. Further research data shows that compounds from OP, NP, NP_*n*_EO (*n* = 2, 9, 40), and NP_1_EC have estrogenic activities against different living organisms [63–67]. In investigation of endocrine disruption effect mixtures of cationic, anionic, nonionic surfactants and some of their degradation products were also tested. Parent compounds have no estrogenic properties for tested organism but for their degradation products (OP, NP, NP_1_EC, NP_2_EC, and NP_2_EO) were observed positive results [68].

## 5. Impact of Surfactants on Abiotic Part of Environment

In previous section of article information about sorption and degradation processes of surfactants in wastewater treatment plants or in the environment is presented. Herein, other important aspects of impact of compounds from the group of surfactants on different part of abiotic environment will be reviewed. So far, an atmospheric input of compounds from the group of surfactants to aquatic environment has not been considered yet. However, evaporation processes of semivolatile compounds (anionic and nonionic surfactants or product of their degradation) from surface waters, soils and vegetation have been recognized as a significant source for such contaminants in the atmosphere [69].

Compounds from the group of SAAs are able to interact on processes that occurred at different interfaces. They can also have influence on processes between air and water interfaces (e.g., suppression of evaporation processes, modification of the surface temperature field during free surface flows, inflection on gas transport and reduction of momentum transport from air to water, and damping of surface waves). Rain droplets contain surfactants monolayer is one of the example mentioned type of interfaces. But processes in this area are still under investigation. Nowadays, scientists believe that there are two mechanisms of surfactant amount reduction on a water surface. First, rain drop can split into many small drops covered with surface active agents. These drops can be transported away from the surface of water by moving air masses. Secondly, bubbles can be formed during rain drop impacts and they will move to water surface, burst, and lead to transporting pollutants to the air [70–74].

Moreover, the occurrence of surfactants in abiotic environment might disturb equilibrium of different compartments. Surfactants are able to form films on aqueous surfaces decreasing the surface tension. It potentially hinders water evaporation and gaseous transportation across the aqueous interface. Those compounds can also increase the solubility of organic compounds in the aqueous phase (increasing mobility of toxic agents in different ecosystems). In the environment this specific system can be observed as a thin boundary between the water basin (e.g., ocean) and the atmosphere, named as sea-surface microlayer (SML). The formation of SML is complicated and it is unknown which physical and chemical processes have influence on migration of chemical compounds in this boundary. For several times occurrence of surface active agents in SML has been proved. Research in this area has shown that SAAs (consist of low-molecular-weight carbonyl compounds) can be also generated in microlayer by microorganisms. In surface water those compounds are produced photochemically from the degradation of refractory dissolved organic matter (e.g., humic substances) and they are taken up quickly by microorganisms [75–77].

Accumulation of surfactants at surface of water can be toxic for marine and freshwater organisms. Some of compounds from the group of surfactants can lead to chronic toxicity or estrogenic responses towards aquatic species [54, 78–82]. At the sea surface surfactants play a role in the recycling and long-range transport of pollutants via marine aerosols. Heavy metals (as pollutants from crustal and urban sources) enriched on the sea surface were found to interact with the surface-active organic matter and become transferred into marine spray [83–85].

The early studies of surfactants in rain water and atmospheric aerosols found that their concentrations were too low to have any effect upon the cloud physical process at high dilution [86]. More concentrated surface active substances or in different mediums may influence the state of the gas-liquid interfaces of atmospheric particles and droplets. Models of cloud formation based on laboratory research suggest that organic compounds significantly provide a decries in surface tension (determining droplet population). Surface tension, which is one of the factors that controls the vapor pressure of small droplets, is the consequence of intermolecular attractive forces tending to minimize the surface area of the liquid [87]. Some compounds from the group of surfactants found in tropospheric aerosol can affect the formation and development of clouds. They might participate in generation of more cloud water due to the reduction of surface tension in a droplet and behave like cloud condensation nuclei (CCN) in the atmosphere. The amount of CCN increases the albedo effect and influences climate change in certain areas (due to relation with cooling effect in the atmosphere) [85, 88–90].

As a result of the intensification of certain types of human activity an upward trend is observed in the content of surfactants in the environmental compartments. Due to their widespread use and freely migration between phases, surfactants and products of their degradation have been detected at various concentrations in different part of abiotic and biotic environment. The occurrence of surface active agents was confirmed in atmospheric precipitation and deposits, surface waters, sediments, soils, and living organisms. Compounds from the group of surfactants have been detected in samples (abiotic: air, snow, lake water, and sediment; biotic: marine and terrestrial organisms) from the areas of residence and economic exploitation by humans and from remote regions like the Antarctic as well. Antarctic ecosystems are sensitive to anthropogenic modifications and highly susceptible to human impact. The global source refers to long-range atmospheric transportation of pollutants (e.g., anionic surfactants) from lower latitude area, but their transport pathways are not well understood [91].

Thus, it is widely accepted that SAAs play an important role of anthropogenic pollutant emission, having versatile environmental consequences. On the other hand, review of the literature also shows that we are far from understanding the environmental fate of surfactants. There is lack of data on composition, properties, and behavior of the organic material (especially surfactants) in the atmosphere or other environmental compartments [1, 92]. The analysis of atmospheric precipitation and deposits is one of the important aspects of the assessment of the degree of environmental pollution caused by surfactants. Both atmospheric precipitation and deposits are considered as a major diffuse source of various types of contaminants. Compounds released into the atmosphere are present in the gaseous phases, in the aerosol phases, or are adsorbed on surface of suspended particles. Two major deposition mechanisms of those pollutants are distinguished, that is, wet (with rain and snow) or dry removal. Deposition is controlled by the distribution of chemical compounds between different phases and their physicochemical properties. On the other hand, the strong dependence was observed between SAAs concentration and deposition on the existing emission background and meteorological conditions, particularly wind direction, sunlight (photodegradation), rain/snowfall, and so forth [92]. Therefore, it is essential to keep control of the content of those compounds in specific environmental samples (e.g., atmospheric precipitation and deposits) and identify parameters that may affect content of surfactants in the different ecosystems.

## 6. Conclusions

Compounds from the group of surfactants are widely applied in different areas of human activities and after use they are discharging to wastewater treatment plant. After appropriated processes (sorption, degradation), they can be emitted to surface waters with effluent streams. In aquatic ecosystems surfactants and their degradation products undergo similar processes as in WWTPs. Compounds from the group of SAAs can also affect living organisms and abiotic parts of the environment. They can be toxic for different types of organisms or disturb their endocrine balance. Moreover, they can interact with different interfaces (water-air, soil/sediment-water) and change natural processes in those systems.

During last decades in environmental samples different types of surfactant were determined using analytical protocols depending on what information is required (total concentration of surfactants or individual analytes from appropriated group of SAAs compounds). But it is still imperative to develop new analytical procedure for investigation analytes from the group of SAAs that occurred in ecosystems to make them easier, less cost-consuming, and more safe for biotic and abiotic elements of the environment. Research data confirmed that they are able to spread between waters, soils, atmosphere, and living organisms from different geographic regions. But we are far from understanding migration pathways and their behavior of those pollutants, their impact on different ecosystems and living organisms. There is a need to investigate those processes in environment to protect abiotic and biotic part of the environment.

## Figures and Tables

**Table 1:** The areas of surfactants application [93–97].

Type of surfactants		
Cationic	Anionic	Nonionic
(i) Disinfectants and antiseptic agents (ii) Ingredient of cosmetics, medicine, laundry detergents (iii) Fabric softeners (iv) Antistatic agents (v) Corrosion inhibitors (vi) Flotation agents	(i) Household detergents and surface cleaners (ii) Shampoos (iii) Hand dishwashing liquids (iv) Laundry detergents (v) Personal care products (vi) Optical brighteners (vii) Dyes (viii) Dispersant, wetting, and suspending agents (ix) Ingredient of pesticides and pharmaceutical products	(i) Household and industrial detergents (ii) Emulsifiers, wetting and dispersing agents (iii) Cleaning products (iv) Cosmetics (v) Paints (vi) Preservative coatings (vii) Ingredient of petroleum products (viii) Ingredient of pesticides (ix) In textiles, pulp, and paper industry

**Table 2:** Sorption percentages or occurrence of surfactants in environmental samples.

Type of analyte	Type of sample	Sorption percentages (%) or concentration	The literature
CSAA			
DTDMAC	Solids/river water	Higher in solid	[98]
CTAB	Solid	~87	[99]

ASAA			
AES	Suspended solids/river water	5–19	[13]
LAS	Suspended solids/river water	<3	
	Suspended solids/estuaries water	11–59	[23]
	Suspended solids/sea water	30–59	
C_10_LAS	Liquid/solid	Higher in water	[14]
C_13_LAS	Solid/liquid	Higher in solid	
SPC	Solid	<1	[100]
Short-chain SPC	Liquid/solid	Higher in water	[14]

NSAA			
AEO	Particulate matter	65–100	[13]
NPEO	Estuaries	25–75	[101]
NPE_1-2_O, NP	Sediment and suspended solids/liquid	Higher in solids	[14]
NPEC	Liquid/solid	Higher in water	

**Table 3:** Research data of aerobic biodegradation assay of selected surfactants.

Type of analytes	Type of sample	Degradation product	Half life or removal efficiency (%/h)	The literature
C_18_TMAC	Activated sludge		50/2.5	[102]
C_16_DMAC	Sea water		50/>360	[103]
QAC		Trimethylamine, dimethylamine, and methylamine	50/72–192	
DT, TT, HT BDD; BDT BDH			50/72–120 50/96–192 50/>360	[20]
DEEDMAC	Sludge, soil, and river water		99/24	[104]

LAS	Surface waters	Mono- and dicarboxylic SPC	50/10–15 h —/96–120 —/168/25°C —/600/13°C	[24, 105]
			50/24 70–90/168	[106]
			56–76/720	[107]
	Sludge amended soils		50/168–792	[10, 108–110]
	Wastewater			
	Wastewater		72.2–100/—	[31]
AES		Alcohols, aldehydes, fatty acids, and sulfur	75.1–98.2/—	[31, 111]
SAS			63.5–93.3/—	[29]
NPEO		NPEC, NP, OP, APDECs	50–80/96–576	[32–34]

NPE_9_	River water River sediment		—/2304 —/<240	[35]

NPE	River water	NPE_2_, NPE_1_, NPEC_1_, and NPEC_2_	68/720/7°C 96/720/25°C	[112]
AEO			79.4–99.7/—	[31]
	Fresh water, activated sludge	Fatty acids, polyethylene glycols	64–~100/72–96	[18, 39, 113–116]
Branched AEO		CAEOs	44/720	[24]

TMAC: tetradecyl trimethyl ammonium chloride; DMAC: dodecyl trimethyl ammonium chloride; QAC: quaternary ammonium compounds; DT: dodecyl trimethyl ammonium bromide; TT: tetradecyl trimethyl ammonium bromide; HT: hexadecyl trimethyl ammonium bromide; BDD: dodecyl benzyl dimethy ammonium bromide; BDT: tetradecyl benzyl dimethyl ammonium chloride; BDH: hexadecyl dimethyl ammonium chloride; DEEDMAC: diethyl ester dimethyl ammonium chloride; LAS: linear alkylbenzene sulfonates; AES: alkyl ethoxysulfates; SAS: secondary alkane sulfonate; NPEC: nonylphenoxy-monocarboxylates; NP: nonylphenol, OP: octylphenol; APDEC: alkylphenol diethoxycarboxylates; CAEO: alkylcarboxylate metabolites; AEO: alcohol ethoxylates.

**Table 4:** The levels of different classes of surfactants in solid and liquid environmental samples (prepared on data published in [1, 3, 117–119] and laboratory testes).

Type of analytes		Type/source of sample	Range of concentration
Soils (*μ*g/kg)			
Anionic	LAS, SPC, PFOA, PFOS	Forest area	3.28–50000
	Total	Soil surface	330 ± 170^a^
Nonionic	AEO	Urban area	69–329
	NPEO, OPEO, NP, OP	Forest area	87–500
	OPEO, NP, OP	Agricultural area	200–229000

Dusts (*μ*g/kg)			
Anionic	LAS	Indoor dust (public buildings)	0.5–1500000
		Street dust	5.0–7.7

Sediments (mg/kg)			
Cationic	Total	River, marine sediment	5–50
	DDAC, BAC, ATAC, DTDMAC		n.d–42300
Anionic	LAS, AES, AS TPS, PFOA, PFOS	Lake, river, and marine sediment	0.0002–3.4
	PFOA, PFOS	Wetland	n.d.–0.0307
Nonionic	NPE, NP, OP, NPEO, OPEO, AEO, NPEC	River, lake, and marine sediment	<MDL–1170

Sewage sludge (mg/kg)			
Cationic	DTDMAC, DHTDMAC	Germany, Switzerland	150–5870
Anionic	LAS, CDEA, SAS, AES, AS, PFOA, PFOS	Germany, Spain, and Switzerland	n.d.–7510
Nonionic	NPEO, AE, NPEC, NP, OP, PEG	USA, Canada, Germany, and Spain	n.d.–601

Sludge (mg/kg)			
Cationic	DDAC, BAC, ATAC	Austria	n.d.–3.6
Anionic	PFOA, PFOS	China, USA	n.d.–7.3
Nonionic	NPEO, OPEO, AE, NPEC, NP	France, Spain, and USA	n.d.–654

Atmospheric waters (*μ*g/L)			
Cationic	Total	Atmospheric water	1.0–11.7^b^
		Aerosols	26.1–129.6^b^
Anionic	Total	Aerosols	14.9–229.1^b^
		Atmospheric water, and cloud water	n.d.–932.2^b^
	PFOA, PFOS	Rain, snow, and street runoff	0.0001–0.182
Nonionic	NP	Rain, snow	n.d.–0.95
Cationic	Total	Dew (urban/rural area)	270/230^c^
		Hoarfrost (urban/rural area)	280/240^c^
Anionic	Total	Dew (urban/rural area)	320/300^c^
		Hoarfrost (urban/rural area)	440/280^c^
Nonionic	Total	Dew (urban/rural area)	770/740^c^
		Hoarfrost (urban/rural area)	990/830^c^

Ground/well/mineral water (*μ*g/L) (for PFOA and PFOS—(ng/L))			
Cationic	Total	Ground water	500–1300
Anionic	DATS, PFOA, PFOS	Ground, raw, and tap water	n.d.–133
	Total	Tap, mineral, and well water	20–193
Nonionic	NPEO, OPEO, NPEC, OPEC, NP, OP	Ground, tap, raw water	n.d.–100

Surface water (*μ*g/L) (for PFOA and PFOS—(ng/L))			
Cationic	Total	Sea water	11–210^d^
	DTDMAC, DEEDMAC, DEQ, BAC, DTMABr, DDABr, DBDMAC, ATAC, DDAC	Surface, river, and sea water	n.d.–75
Anionic	Total	River water, and sea water	5–150 (5–360^e^)
	LAS, AES, AS, SPC, DATS, PFOA, PFOS	River, lake, and sea water	n.d.–2210000
Nonionic	Total	River water	27–222
	NPEO, OPEO, C_12–16_EO, NPEC, OPEC, NP, OP	River water, lake water	n.d.–14

Wastewaters (*μ*g/L)			
Cationic	Total	China	374–2116
	DDAC, BAC, ATAC, CTAB	Austria, Algeria, Spain, and USA	n.d.–49
Anionic	Total	Canada	120–9340
	LAS, PFOA, PFOS	Germany, Austria, Spain, Japan, and China	0.0087–1630
Nonionic	Sum	China	374–2116
	NPEO, OPEO, C_12–16_EO, NP, OP, PEG, NPEC, OPEC	USA, Spain, France, Austria, Italy, Canada, Russia, and Japan	n.d.–2340

Sewage (*μ*g/L)			
Cationic	DTDMAC, DEEDMAC DEQ	Germany	n.d.–140
Anionic	LAS, DATS	Italy	0.52–2360
Nonionic	NPEO, NP		0.3–208

^a^(*μ*M/kg); ^b^(pmol/m^3^); ^c^mean value; ^d^(*μ*mol/L); ^e^(pmol/L).

**Table 5:** Toxicity for different classes of surfactants on different test organisms [8].

Test organism	Analyte	Parameter	Mean value (mg/L or mg/kg)
Aquatic organisms			
*Daphnia magna *	DEEDMAC	LC_50_/24 h	14.8
	TMABr	IC_50_/24 h	0.14
	BDMAC		0.13
	LAS		1.22–13.9^f^
	NPEO_9_	LC_50_/48 h	14
	NP		0.19
*Dunaliella* sp. (green alga)	TMAC	EC_50_/24 h	0.79
	LAS		3.5
*Salmo gairdneri* (rainbow trout)	TMAC		1.21
	LAS		3.63
	AS		33.61
	AES		10.84
	C_12_EO_6_		22.38
	OPEO_6_		6.44
*Gambusia affinis* (mosquito fish)	TMAC		8.24
	AS	EC_50_/48 h	40.15
	AES		13.64
	C_12_EO_6_		29.26
	OPEO_6_		9.65
*Carassius auratus* (gold fish)	TMAC		3.58
	AS		38.04
	AES		12.35
	C_12_EO_6_		28.02
	OPEO_6_		9.24

Terrestial organisms			
Bush beans, radish, and grasses		NOEC/76 days	27
Potatoes			16
Sorghum	LAS	EC_50_/21 days	167
Sunflower			289
Mung bean			316

^f^Data for different homologues of LAS compounds.

## Abbreviations

AE: Alcohol ethoxylates
AES: Alcohol ethoxysulfates
AEO: Alcohol polyethoxylates
APDEC: Alkylphenol diethoxycarboxylates
APEO: Alkylphenol ethoxylates
AS: Fatty alcohol sulphates
ASAA: Anionic surface active agent
ATAC/ATABr: Alkyl trimethyl ammonium chloride/bromide
BAC: Benzalkonium chloride
BDD: Dodecyl benzyl dimethy ammonium bromide
BDH: Hexadecyl dimethyl ammonium chloride
BDT: Tetradecyl benzyl dimethyl ammonium chloride
C_12−16_EO: Alkyl ethoxylates
CD: Conductivity detection
CDEA: Alkyl carboxylated metabolites
CCN: Cloud condensation nuclei
CSAA: Cationic surface active agent
CTAB: Cetyl trimethyl ammonium bromide
DATS: Dialkyltetralin sulphonates
DBDMAC: Dodecyl benzyl dimethyl ammonium chloride
DDAC: Dodecyl ammonium chloride
DDABr: Dodecyl ammonium bromide
DEEDMAC: Diethyl ester dimethyl ammonium chloride
DEQ: Diesterquaternary
DHTDMAC: Dihydrogenated tallow dimethyl ammonium chloride
DiSB: Disulfine blue
DMAC: Dodecyl trimethyl ammonium chloride
DMABr/DT: Dodecyl trimethyl ammonium bromide
DTDMAC: Ditallow dimethyl ammonium chloride
EC: Effective concentration
EDC: Endocrine disruptor compounds
ELS: Evaporative light scattering detector
FLD: Fluorescence detector
GAC: Green analytical chemistry
GC: Gas chromatography
HPLC: High performance liquid chromatography
HT: Hexadecyl trimethyl ammonium bromide
IC: Inhibitory concentration
LAS: Linear alkylbenzene sulfonates
LC: Lethal concentration
MB: Methylene blue
MS: Mass spectrometry
NOEC: No observed effect concentration
NP: Nonylphenol
NPEC: Nonylphenoxy-monocarboxylates
NPE/NPEO: Nonylphenol ethoxylates
NSAA: Nonionic surface active agent
OPEC: Octylphenoxy-monocarboxylates
PEG: Polyethylene glycol
PFOA: Perfluorooctanoic acid
PFOS: Perfluorooctane sulfonate
SAA: Surface active agents
SAS: Secondary alkane sulfonate
SDS: Sodium dodecyl sulfate
SML: Sea-surface microlayer
SPC: Carboxylic sulfophenyl acids
TMAC: Tetradecyl trimethyl ammonium chloride
TMABr/TT: Tetradecyl trimethyl ammonium bromide
TPS: Tetradecyl dimethyl ammonium propanesulfonate
QAC: Quaternary ammonium compounds
UV: Ultra-violet detector
WWTP: Wastewater-treatment plants.
